# Association between nocturia frequency and depression in adults: evidence from NHANES 2021–2023 and a Chinese exploratory cohort

**DOI:** 10.3389/fpubh.2026.1801592

**Published:** 2026-10-07

**Authors:** Cong Cheng, Yichi Yang, Tao Yang, Jinmin Zeng, Youji Yan, Yixiang Liao

**Affiliations:** 1 Department of Urology, Jingzhou Hospital Affiliated to Yangtze University, Jingzhou, Hubei, China; 2 Department of Cardiology, Xiantao First People's Hospital, Xiantao, Hubei, China

**Keywords:** age, cross-sectional study, depression, NHANES, nocturia

## Abstract

**Background:**

Nocturia and depression commonly coexist and may share underlying pathways. We examined the cross-sectional association between nocturia frequency and clinically relevant depressive symptoms.

**Methods:**

We analyzed 4,162 adults from the National Health and Nutrition Examination Survey (NHANES) 2021–2023. Nocturia was categorized as 0, 1, 2, or ≥3 voids/night; a Patient Health Questionnaire-9 (PHQ-9) score ≥10 defined clinically relevant depressive symptoms. Survey-weighted logistic regression, an ordinal trend test, and subgroup analyses were conducted. A Chinese cohort (*n* = 255) was used for exploratory sleep-pathway analyses. A sensitivity analysis used a modified Pittsburgh Sleep Quality Index (PSQI) score excluding the sleep-disturbance component, which includes the nocturnal bathroom-use item.

**Results:**

In the fully adjusted complex survey model, two voids/night (OR: 1.42; 95% CI: 1.05–1.92; *p* = 0.026) and ≥3 voids/night (OR: 1.83; 95% CI: 1.40–2.40; *p* < 0.001) were associated with higher odds of clinically relevant depressive symptoms, with an ordinal trend (*p* < 0.001). An exploratory age interaction was observed (Wald *p* for interaction = 0.043). In the Chinese exploratory cohort, the unstandardized indirect effect through total PSQI score was 0.244 (bootstrap 95% CI 0.094–0.426), whereas the total and direct effects were non-significant. A sensitivity analysis excluding the PSQI sleep-disturbance component yielded a smaller but still positive indirect effect (0.181; bootstrap 95% CI 0.053–0.342).

**Conclusions:**

Frequent nocturia was cross-sectionally associated with clinically relevant depressive symptoms. The Chinese exploratory outpatient cohort provided exploratory support for a sleep-related statistical pathway, which was attenuated but remained positive after excluding the PSQI sleep-disturbance component. These findings highlight nocturia as a clinically relevant symptom linking urinary, sleep, and mental health, while longitudinal studies are needed to clarify temporal and causal relationships.

## 1 Introduction

Nocturia, defined as waking at night to urinate at least once, is a prevalent lower urinary tract symptom (LUTS) (1, 2), affecting 50 million people in the United States, with 10 million diagnosed with nocturia but only 1.5 million receiving specific therapy (3). Its multifactorial etiology encompasses urological conditions such as benign prostatic hyperplasia and overactive bladder, systemic diseases including diabetes and heart failure, and lifestyle factors like excessive fluid intake and irregular sleep patterns (4–6). Beyond its urological implications, nocturia disrupts sleep continuity, leading to fatigue, reduced daytime functioning, impaired quality of life, depression, and significant healthcare costs (7–9). Among older adults, nocturia is associated with falls, fractures, and increased mortality, underscoring its importance as a public health concern (10). Nationally representative data from the National Health and Nutrition Examination Survey (NHANES) provide robust opportunities to investigate nocturia's epidemiology and correlates in the U.S. population, using validated self-reported measures such as the nocturia frequency question (KIQ026). Despite the substantial burden of nocturia, its psychological consequences, particularly its association with depression, remain insufficiently explored in large-scale population-based studies, particularly using recent NHANES cycles.

Depression represents a leading cause of disability and disease burden worldwide (11), affecting approximately 280 million individuals globally and exhibiting a lifetime prevalence of 7.8% in the in the United States. Characterized by persistent sadness, anhedonia, and cognitive impairment, depression imposes significant personal, social, and economic costs, including increased healthcare utilization, reduced productivity, and elevated risk of chronic diseases (12, 13). Risk factors for depression encompass genetic predisposition, chronic psychosocial stress, and medical comorbidities, with sleep disturbances emerging as a critical mediator in the pathogenesis of depressive symptoms (13). The NHANES utilizes the Patient Health Questionnaire-9 (PHQ-9), a validated screening tool (14), where a score of ≥10 indicates moderate-to-severe depression risk (15). Epidemiological data reveal higher depression prevalence among women, older adults, individuals with lower socioeconomic status, and those burdened by chronic illnesses (16). Given nocturia's potential to fragment sleep and induce distress, it is biologically plausible that nocturia may contribute to depressive symptoms, particularly among vulnerable populations.

However, the relationship between nocturia and depression remains incompletely defined, with existing studies offering limited insights into nocturia-specific effects, frequency thresholds, and potential effect modification by age. Previous research demonstrated that LUTS, encompassing nocturia, were significantly associated with increased depression risk (17–19). Park et al. (20), analyzing NHANES 2005–2008 data, identified a positive association between LUTS and depression (OR 5.89; 95% CI 3.44-10.11), but did not focus specifically on nocturia, nor did they conduct age-stratified analyses. Furthermore, few investigations have leveraged recent NHANES cycles (2021–2023). The role of age as a potential effect modifier is a crucial gap, given that younger and older adults may experience nocturia differently due to distinct etiologies and varying levels of psychological resilience.

This study aims to address these gaps by examining the association between nocturia frequency (0, 1, 2, or ≥3 nocturnal voids per night) and depression risk (PHQ-9 ≥10) in a nationally representative sample from NHANES 2021–2023, with specific focus on age-stratified effects. Furthermore, a separate Chinese exploratory outpatient cohort was incorporated to complement the assessment of associations among nocturia frequency, sleep quality, and depressive symptoms.

## 2 Methods

### 2.1 Participants from NHANES 2021–2023 and eligibility criteria

Data for this study were derived from the National Health and Nutrition Examination Survey (NHANES, https://www.cdc.gov/nchs/nhanes/) conducted between August 2021 and August 2023. NHANES is a nationwide program integrating interviews, physical examinations, and laboratory tests to assess the health and nutritional status of adults and children in the United States. During this period, a total of 11,933 participants were enrolled in NHANES. According to the NHANES Kidney Conditions Questionnaire documentation, the nocturnal voiding item (KIQ481) was administered to participants aged ≥20 years; therefore, the eligible starting population for the present analysis was restricted to adults aged ≥20 years who were eligible for KIQ481.

Participants were excluded if they had missing data for KIQ481 (number of times waking to urinate at night), age, or the complete Patient Health Questionnaire-9 (PHQ-9). Additional exclusion criteria included missing information on education, marital status, family income-to-poverty ratio (PIR), body mass index (BMI), responses to whether they had smoked at least 100 cigarettes in their lifetime, responses to whether a doctor or health professional had ever diagnosed them with weak or failing kidneys (excluding kidney stones, bladder infections, or urinary incontinence), responses regarding diabetes diagnosis, self-reported sleep duration on weekdays and weekends, or laboratory results for high-sensitivity C-reactive protein (hs-CRP).

After applying these criteria, a total of 4,162 participants were included in the final analysis. Figure 1 presents the detailed flowchart of participant selection.

**Figure 1:**
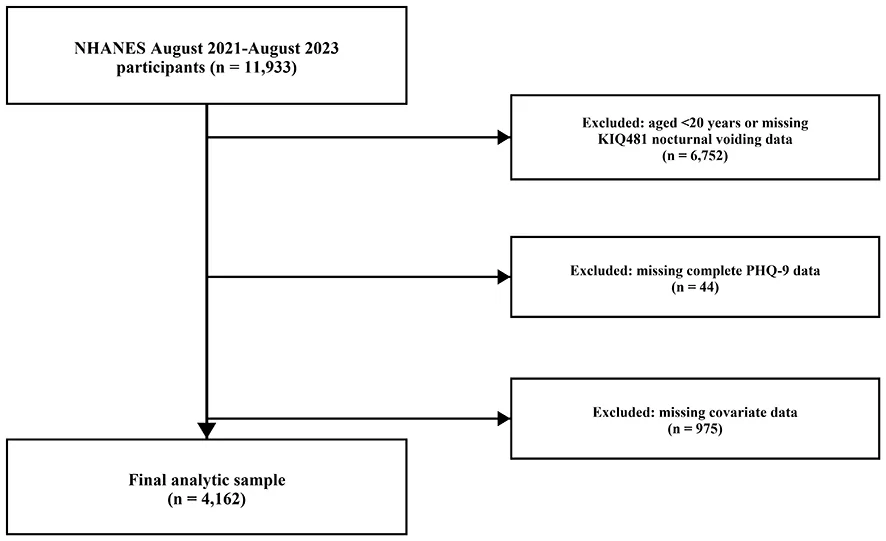
Flowchart of participant selection from the NHANES 2021-2023 sample.

### 2.2 Frequency of nocturnal voiding

Information on nocturia frequency was obtained from the Kidney Conditions Questionnaire. Participants were categorized based on their response to the question: “In the past 30 days, during a typical night, how many times do you wake up to urinate?” Participants were classified into four groups: Q1 (0 nocturnal voids), Q2 (1 void), Q3 (2 voids), and Q4 (3 or more voids).

### 2.3 Definition of depression

Depressive symptoms were assessed using the Patient Health Questionnaire-9 (PHQ-9), a validated screening instrument comprising nine questions (14). Each question has four response options: “not at all,” “several days,” “more than half the days,” and “nearly every day,” scored from 0 to 3, respectively. The total PHQ-9 score ranges from 0 to 27, reflecting the severity of depressive symptoms experienced over the previous 2 weeks. A total score of ≥10 was defined as indicative of depression, consistent with a sensitivity and specificity of 88% for detecting major depressive disorder.

### 2.4 Covariates

Covariates included demographic, questionnaire, and laboratory data such as age, race, marital status, education, family income, smoking status, alcohol consumption, BMI, diabetes status, history of weak or failing kidneys, sleep duration, and hs-CRP levels.

Age was categorized into three groups: < 45 years, 45–64 years, and ≥65 years. Race was classified as Mexican American, Non-Hispanic White, Non-Hispanic Black, Other Hispanic, and Other Race. Education was categorized as less than 9th grade, 9th−11th grade, high school graduate, some college or associate degree, and college degree or above. Marital status was classified as married/living with a partner, widowed/divorced/separated, or never married. PIR was divided into three categories: < 1.5, 1.5–3.5, and ≥3.5 (21). BMI was used to assess obesity status and categorized as < 25, 25–30, and ≥30 kg/m^2^. Alcohol consumption was classified as no (participants who consumed fewer than 12 alcoholic drinks in the past 12 months) and yes (participants who consumed at least 12 alcoholic drinks in the past 12 months). Smoking status was categorized as no (fewer than 100 cigarettes smoked in a lifetime) and yes (at least 100 cigarettes smoked in a lifetime) (22). Diabetes status was classified as yes, no, or borderline. Weekday and weekend sleep duration were categorized as < 6 h, 6–7.5 h, 7.5–9 h, and ≥9 h. hs-CRP levels were divided into tertiles after ranking: 0.11–1.05 mg/L, 1.06–3.17 mg/L, and 3.19–110.98 mg/L. These categories were used for descriptive and subgroup analyses; in multivariable regression models, age, PIR, BMI, sleep duration, and hs-CRP were entered as continuous covariates unless otherwise specified.

### 2.5 Chinese exploratory outpatient cohort

A separate hospital-based outpatient cohort recruited 259 eligible and willing adults at the urology outpatient clinic of Jingzhou Central Hospital, China, from September 2024 to May 2025. Eligible participants were aged ≥18 years and presented for evaluation or consultation for lower urinary tract symptoms or other urological complaints, such as urinary frequency, urgency, dysuria, hematuria, urinary incontinence, lower abdominal or pelvic discomfort, prostate-related symptoms, or urolithiasis-related consultation. Participants volunteered to take part, provided written informed consent, and were able to understand and complete the Chinese questionnaires. Recruitment was not restricted to nocturia or to a single urological diagnosis; nocturia status was classified according to self-reported nocturnal voiding frequency, allowing inclusion of participants with and without nocturia. To support the exploratory analysis of nocturia, sleep quality, and depressive symptoms, participants were required to have questionnaire information on nocturnal voiding frequency, the PHQ-9, and the Pittsburgh Sleep Quality Index (PSQI). Individuals were excluded if they were unable to provide informed consent or complete the questionnaires because of cognitive or communication limitations, were pregnant, had current urinary catheterization, had undergone recent urological surgery, had clinically apparent acute urinary tract infection or gross hematuria at recruitment, or had acute severe illness requiring emergency treatment or hospitalization. Four participants had missing nocturia data; therefore, 255 participants were included in nocturia-group comparisons.

### 2.6 Statistical analysis

The NHANES analytic dataset was constructed by merging relevant official NHANES data files according to the unique respondent sequence number (SEQN). Survey analyses incorporated the mobile examination center (MEC) weight (WTMEC2YR), masked variance pseudo-strata (SDMVSTRA), and pseudo-primary sampling units (SDMVPSU) from the official demographic file to account for the complex survey design. Continuous variables were expressed as weighted mean +/− standard deviation, while categorical variables were presented as weighted percentages. Group differences were assessed using weighted chi-square tests for categorical variables.

Logistic regression analyses were performed to estimate the association between nocturia frequency and clinically relevant depressive symptoms, expressed as odds ratios (ORs) with 95% confidence intervals (CIs). Model 1 was unadjusted; Model 2 adjusted for age, race, and sex; and Model 3 adjusted for all prespecified covariates. For the formal trend test, the four nocturia categories were entered as an ordinal variable (0–3). Figure 2 presents model-based estimates across these categories.

**Figure 2:**
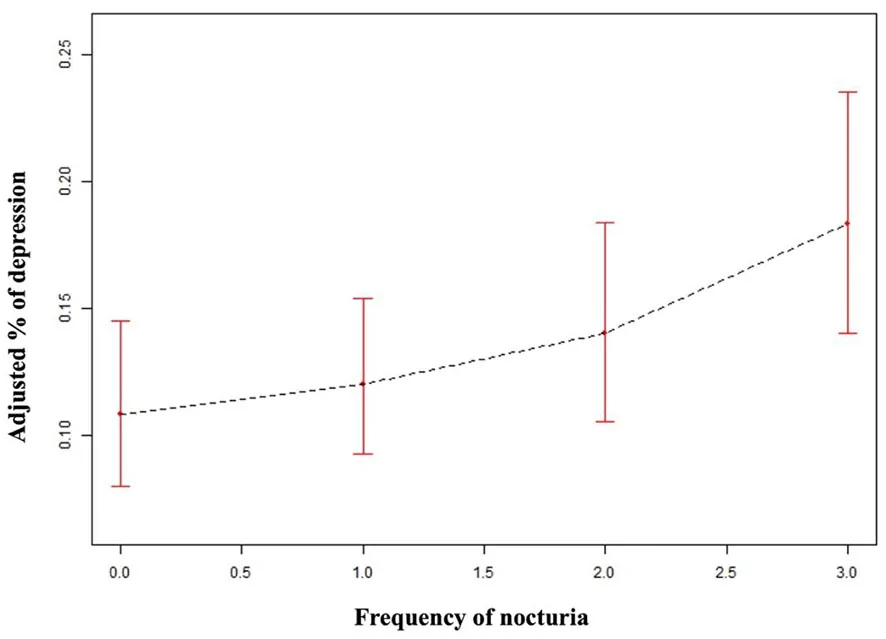
Model-based association between ordinal nocturia-frequency categories and clinically relevant depressive symptoms.

Subgroup analyses were conducted to examine the association between nocturia frequency and depression stratified by age, gender, race, education, marital status, PIR, BMI, drinking status, smoking status, diabetes status, history of weak or failing kidneys, weekday sleep duration, weekend sleep duration, and hs-CRP levels. In these subgroup and interaction analyses, all covariates except the stratification factor were included in the models.

In the Chinese exploratory outpatient cohort, kernel density estimation was used to visualize the distributions of PSQI and PHQ-9 scores across nocturia frequency subgroups.

Partial correlation analyses were conducted to examine the association between sleep quality (PSQI score) and depressive symptoms (PHQ-9 score) within each nocturia frequency subgroup. Age was included as a covariate to control for its potential confounding effect. Partial correlation coefficients (r) and corresponding 95% confidence intervals were estimated. To account for multiple subgroup tests, *p-values* from the four nocturia-frequency subgroup analyses were adjusted using the Benjamini–Hochberg false discovery rate (FDR) method.

In addition, exploratory indirect-effect analysis was performed to examine indirect associations linking nocturia frequency, sleep quality, and depressive symptoms. The total Pittsburgh Sleep Quality Index (PSQI) score was retained as the primary exploratory sleep-quality measure because it represents the standard scoring approach for the PSQI. All path coefficients were reported as unstandardized estimates. Indirect, direct, and total effects were estimated using 5,000 bootstrap resamples, adjusting for age. Because the standard PSQI includes a sleep-disturbance item related to getting up to use the bathroom, we conducted a sensitivity analysis using a modified PSQI score calculated from the six PSQI components that do not directly include the sleep-disturbance component. Given the cross-sectional measurement of nocturia frequency, sleep quality, and depressive symptoms, this analysis was defined as an exploratory statistical pathway analysis.

All statistical analyses were conducted using Statistical Package for the Social Sciences (SPSS), version 26.0 for Mac., the “PROCESS” macro in SPSS (version 4.2), R (v4.1.2) and EmpowerStats (version 2.0), with a two-sided *p-value* of < 0.05 considered statistically significant.

## 3 Results

### 3.1 Characteristics of participants from NHANES 2021–2023

This study included 4,162 participants from NHANES 2021–2023: 858 (20.6%) without nocturia (Q1), 1,597 (38.4%) with one nocturnal void (Q2), 909 (21.8%) with two (Q3), and 798 (19.2%) with three or more nocturnal voids (Q4). Baseline characteristics are presented in Table 1. Apart from gender distribution and drinking, significant differences were observed among the groups. Compared with participants without nocturia, those with three or more nocturnal voids were older; more frequently identified as Non-Hispanic Black or Other Hispanic; had lower educational attainment (high school or below); and were more likely to be widowed, divorced, or separated. They also showed higher smoking rates, lower PIR, higher BMI, greater prevalence of diabetes, and were more frequently informed of weak or failing kidneys. Participants with more frequent nocturia also had higher PHQ-9 scores and higher hs-CRP levels.

**Table 1:** Weighted baseline characteristics of included participants.

Variables	Total (*N* = 4,162)	Q1 (*N* = 858)	Q2 (*N* = 1,597)	Q3 (*N* = 909)	Q4 (*N* = 798)	*p*
**Age (years)**						< 0.001
< 45	43.79	65.06	42.08	28.52	34.74	
45–64	34.34	25.80	36.49	39.33	35.89	
≥65	21.87	9.14	21.43	32.14	29.37	
**Gender**						0.299
Male	49.09	51.85	49.21	46.68	47.58	
**Race**						0.042
Mexican American	6.35	6.73	6.58	6.37	5.25	
Other Hispanic	8.18	7.53	7.40	9.07	9.90	
Non-Hispanic White	64.95	66.02	67.45	63.84	58.98	
Non-Hispanic Black	9.51	7.16	8.62	10.58	13.67	
Other race	11.01	12.56	9.95	10.14	12.21	
**Education**						< 0.001
Less than 9th	2.31	1.63	1.69	2.93	3.99	
9–11th	5.59	3.79	4.55	7.55	8.30	
High school	23.71	18.96	22.32	29.60	26.95	
Some college or AA degree	30.69	29.40	31.62	31.47	29.55	
College or above	37.70	46.22	39.83	28.45	31.21	
**Marital status**						< 0.001
Married/Living with partner	61.76	61.26	67.05	55.99	56.91	
Widowed/Divorced/Separated	18.62	12.23	15.41	27.61	24.83	
Never married	19.62	26.51	17.53	16.39	18.26	
**Poverty-income ratio (PIR)**						< 0.001
< 1.5	20.65	15.73	17.09	25.12	30.67	
1.5–3.5	33.26	30.51	33.53	37.34	31.94	
≥3.5	46.09	53.76	49.38	37.54	37.39	
**BMI (kg/m** ^**2**^ **)**						< 0.001
< 25	26.41	31.24	28.04	21.28	21.62	
25–30	32.62	35.91	33.13	29.15	30.69	
≥30	40.98	32.85	38.83	49.57	47.68	
**Drink**						0.659
Yes	92.05	92.73	92.24	91.06	91.75	
**Smoke**						< 0.001
Yes	37.98	33.44	35.30	44.94	42.68	
**Diabetes**						< 0.001
Yes	10.91	5.63	8.77	14.84	18.86	
No	86.13	92.67	88.28	81.40	77.31	
Borderline	2.95	1.70	2.95	3.77	3.83	
**Weak/failing kidneys**						< 0.001
Yes	2.57	0.89	1.99	3.89	4.81	
**Sleep duration on workdays**						0.004
< 6	8.00	6.84	7.08	10.12	9.35	
6–7.5	28.15	31.80	25.85	30.47	25.60	
7.5–9	44.54	46.63	48.02	38.44	40.51	
≥9	19.31	14.73	19.05	20.97	24.54	
**Sleep duration on weekends**						0.030
< 6	5.11	4.29	4.21	6.80	6.42	
6–7.5	17.63	16.73	17.39	18.42	18.58	
7.5–9	39.26	43.21	42.03	32.10	35.40	
≥9	38.00	35.77	36.38	42.68	39.60	
**PHQ-9 score**	3.90 ± 4.51	3.56 ± 4.13	3.52 ± 4.28	4.18 ± 4.75	4.93 ± 5.07	< 0.001
**hs-CRP (mg/L)**	3.82 ± 7.31	2.99 ± 5.06	3.57 ± 6.98	4.76 ± 8.75	4.51 ± 8.66	0.016

PIR, Poverty-income ratio; Q1, 0 nocturnal voids; Q2, 1 void; Q3, 2 voids; Q4, 3 or more voids.

### 3.2 Association between frequency of nocturia and depression

Partially adjusted models indicated higher odds of clinically relevant depressive symptoms among participants with two nocturnal voids (OR 1.88, 95% CI 1.37–2.58) and ≥3 nocturnal voids (OR 2.35, 95% CI 1.89–2.91) compared with none (Table 2). In the fully adjusted model, the OR was 1.42 (95% CI 1.05–1.92; *p* = 0.026) for two nocturnal voids and 1.83 (95% CI 1.40–2.40; *p* < 0.001) for ≥3 nocturnal voids. Modeling nocturia frequency as an ordinal variable supported an increasing trend (*p* for trend < 0.001; Figure 2).

**Table 2:** Weighted association between frequency of nocturia and depression.

Depression	Q1	Q2	*p*	Q3	*p*	Q4	*p*
Model 1	1 (Ref.)	1.06 (0.75, 1.50)	0.711	1.39 (1.08, 1.79)	0.015	1.77 (1.46, 2.15)	< 0.001
Model 2	1 (Ref.)	1.28 (0.91, 1.81)	0.147	1.88 (1.37, 2.58)	< 0.001	2.35 (1.89, 2.91)	< 0.001
Model 3	1 (Ref.)	1.25 (0.87, 1.79)	0.211	1.42 (1.05, 1.92)	0.026	1.83 (1.40, 2.40)	< 0.001

Q1, 0 nocturnal voids; Q2, 1 void; Q3, 2 voids; Q4, ≥3 voids. Model 1: unadjusted. Model 2: age, sex, and race. Model 3: age, sex, race, education, marital status, PIR, BMI, alcohol use, smoking, diabetes, weak/failing kidneys, weekday and weekend sleep duration, and hs-CRP. Model 3 p for trend < 0.001.

### 3.3 Association between frequency of nocturia and depression in different subgroups

Figure 3 presents subgroup-specific associations between nocturia frequency and clinically relevant depressive symptoms, with no nocturia (Q1) as the reference group. Across most subgroups, the ORs for frequent nocturia (Q4, ≥3 voids/night) were generally above Q1, indicating a directionally consistent positive association. Age-stratified analyses suggested possible heterogeneity across age groups, with exploratory evidence of interaction by age (*p* for interaction = 0.043). Figure 4 shows the smoothed associations between nocturia frequency and depressive symptoms across age groups.

**Figure 3:**
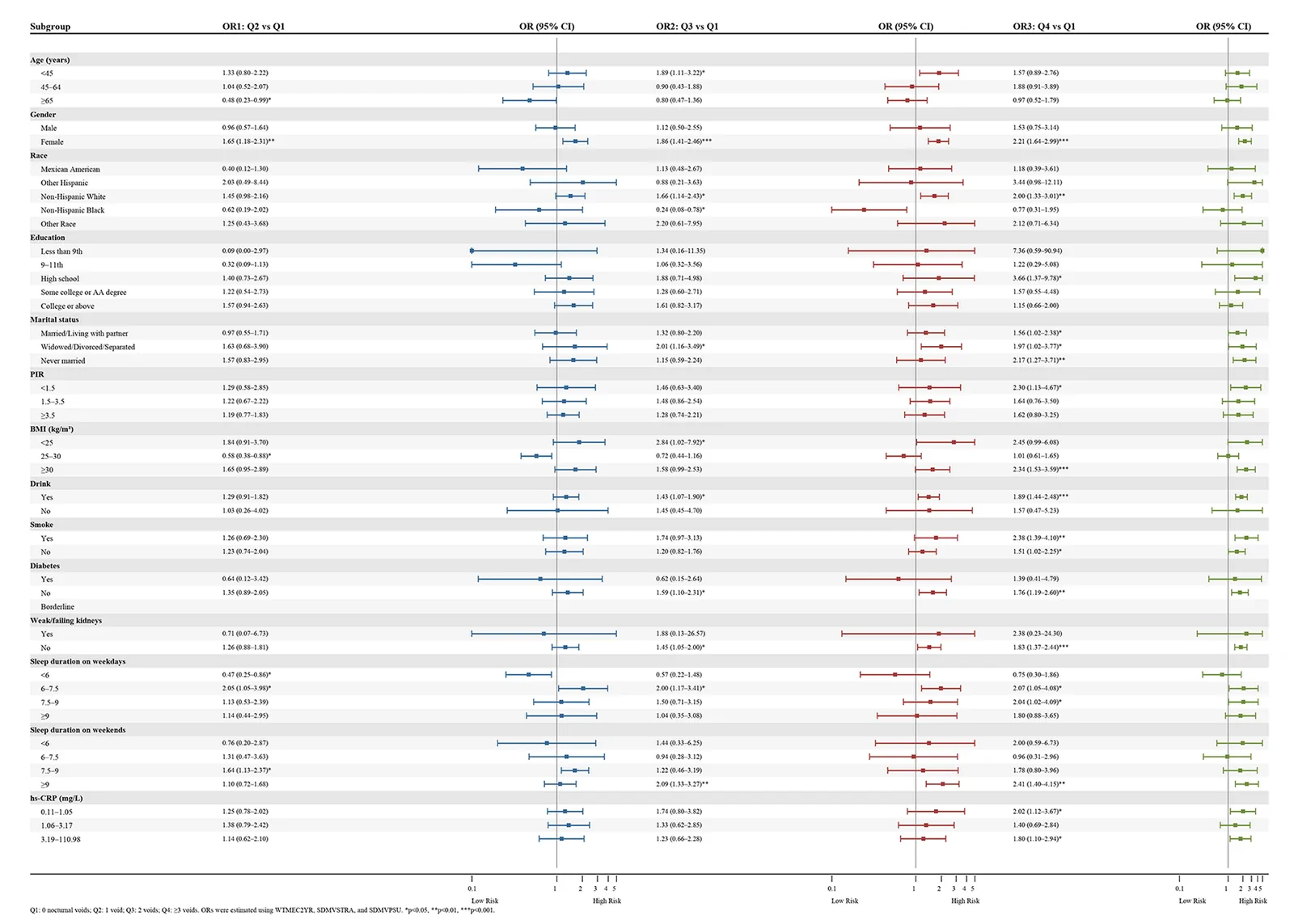
Subgroup-specific associations between nocturia frequency and clinically relevant depressive symptoms.

**Figure 4:**
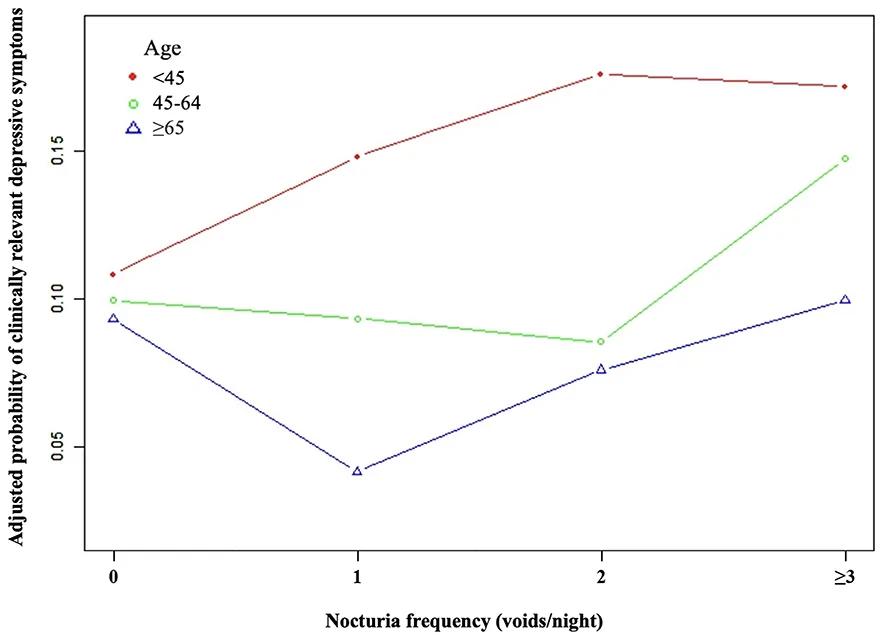
Model-based associations between nocturia frequency and clinically relevant depressive symptoms across age groups.

### 3.4 Chinese exploratory cohort and indirect-effect analysis

#### 3.4.1 Characteristics of the Chinese exploratory cohort

In the Chinese exploratory outpatient cohort (*n* = 259), age and sleep quality differed across nocturia-frequency groups (Table 3). Four participants had missing nocturia data, leaving 255 for group comparisons. Participants with higher nocturia frequency had higher PSQI scores, indicating poorer sleep quality (*p* < 0.001; Figure 5a). However, PHQ-9 scores did not differ significantly across nocturia groups (*p* = 0.176), and their distributions largely overlapped (Figure 5b).

**Table 3:** Characteristics of the Chinese exploratory cohort.

Variables	Total (*N* = 255	V1 (*N* = 100)	V2 (*N* = 57)	V3 (*N* = 57)	V4 (*N* = 41)	*p*
**Age (years)**	36.77 ± 9.84	35.25 ± 9.82	35.74 ± 9.02	37.33 ± 9.60	41.07 ± 10.38	0.011
**Gender**						0.611
Male	66.80	67.00	66.67	73.68	60.98	
**Race**						0.519
Han Chinese	99.00	96.49	98.25	100.00	99.00	
Other race	1.00	3.51	1.75	0.00	1.00	
**Education**						0.176
Less than high school	66.27	74.49	71.93	53.57	60.00	
High school	21.96	16.33	21.05	30.36	25.00	
Junior college	8.63	8.16	5.26	12.50	7.50	
College or above	3.14	1.02	1.75	3.57	7.50	
**Marital status**						0.332
Married/Living with partner	29.18	32.65	35.09	19.30	24.39	
Widowed/Divorced/Separated	25.68	22.45	19.30	31.58	34.15	
Never married	45.14	44.90	45.61	49.12	41.46	
**Drink**						0.926
Yes	65.64	67.00	64.91	64.91	60.98	
**Smoke**						0.428
Yes	43.24	47.00	36.84	40.35	51.22	
**PHQ-9 score**	4.20 ± 3.25	3.75 ± 3.15	4.68 ± 3.39	4.68 ± 3.11	3.85 ± 3.34	0.176
**PSQI score**	4.91 ± 3.14	3.45 ± 2.34	6.07 ± 3.39	5.65 ± 2.77	5.71 ± 3.52	< 0.001

V1, 0 nocturnal voids; V2, 1 void; V3, 2 voids; V4, 3 or more voids.

**Figure 5:**
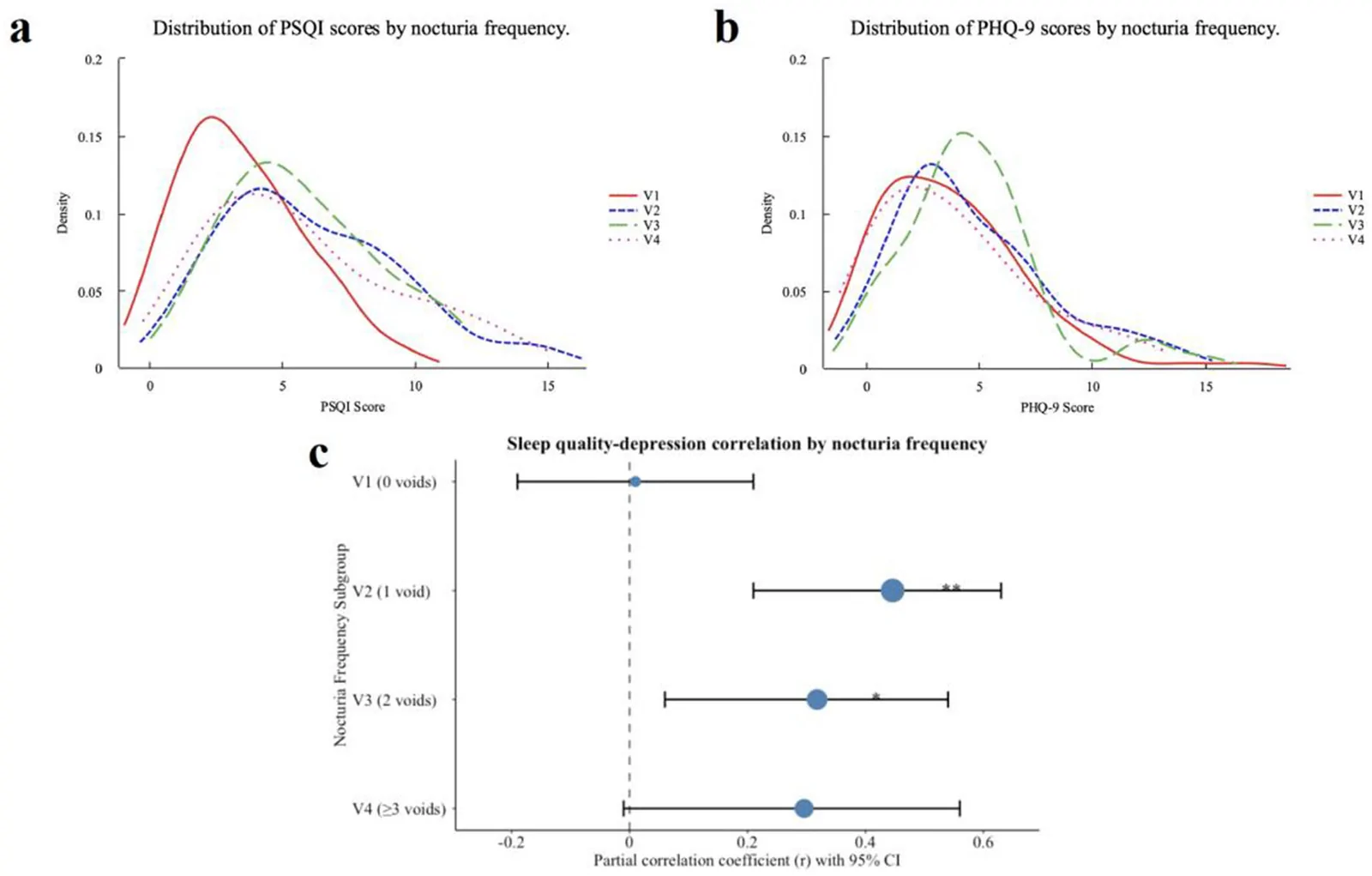
Correlation between sleep quality and depressive symptoms by nocturia frequency subgroups. **(a)** Distribution of PSQI scores by nocturia frequency, **(b)** Distribution of PHQ-9 scores by nocturia frequency, **(c)** Age-adjusted partial correlation between sleep quality and depressive symptoms by nocturia frequency subgroups.

#### 3.4.2 Partial correlation and exploratory indirect-effect analysis

Partial correlation analyses adjusted for age examined the association between sleep quality and depressive symptoms within each nocturia-frequency group. Correlations were observed among participants reporting one (*r* = 0.446, *p* = 0.001; FDR-adjusted *p* = 0.0021) and two (*r* = 0.318, *p* = 0.016; FDR-adjusted *p* = 0.0318) nocturnal voids (Figure 5c). No significant association was observed among participants reporting no nocturia or ≥3 nocturnal voids after FDR correction.

Exploratory indirect-effect analysis using total PSQI score showed that nocturia frequency was associated with higher PSQI scores (unstandardized path *a* = 0.845, *p* < 0.001), and PSQI scores were positively associated with PHQ-9 scores (unstandardized path *b* = 0.289, *p* < 0.001). The unstandardized indirect effect through total PSQI was 0.244 (bootstrap SE = 0.085; 95% CI 0.094–0.426). However, the total effect (*c* = 0.100, *p* = 0.590) and direct effect (*c*′ = −0.144, *p* = 0.445) were non-significant. In sensitivity analysis using a modified PSQI score that excluded the sleep-disturbance component, the unstandardized indirect effect was smaller but remained positive (a × b = 0.181; bootstrap SE = 0.074; 95% CI 0.053–0.342). Given the smaller and relatively younger outpatient sample, together with the non-significant total and direct effects, this finding should be interpreted as exploratory evidence supporting a possible sleep-related statistical pathway rather than as confirmatory mediation of the NHANES association (Figure 6).

**Figure 6:**
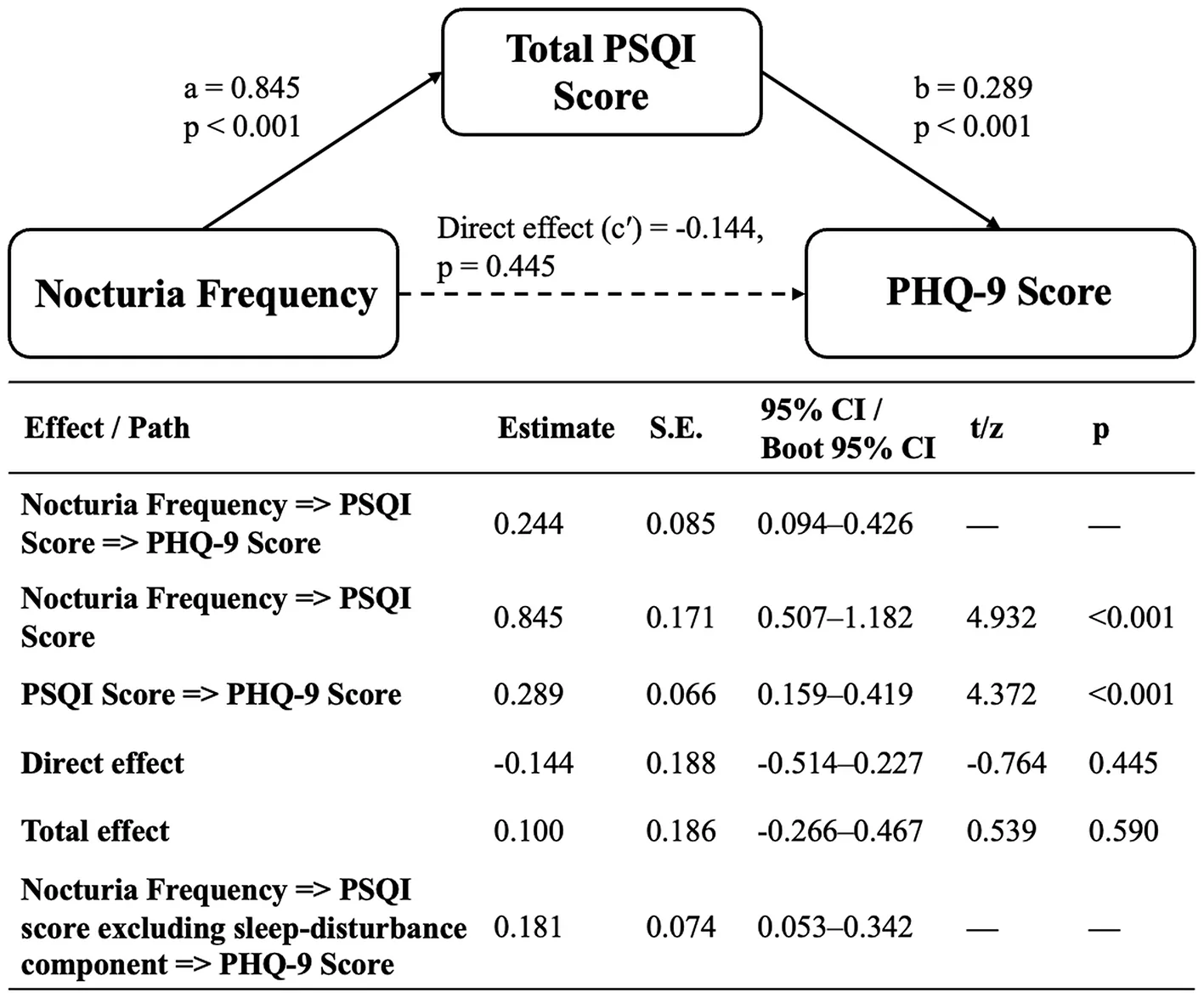
Exploratory sleep-related indirect-effect analysis using nocturia frequency as the exposure in the Chinese outpatient cohort. The total PSQI score was retained as the primary exploratory sleep-quality measure, and the sensitivity analysis used a modified PSQI score excluding the PSQI sleep-disturbance component. Path estimates are age-adjusted unstandardized coefficients. For indirect-effect rows, statistical evidence was assessed using bootstrap 95% confidence intervals; t/z and *p-values* are therefore not reported for these rows. For individual path, direct-effect, and total-effect estimates, t statistics and *p-values* were obtained from age-adjusted regression models.

## 4 Discussion

In this nationally representative study of 4,162 U.S. adults from NHANES August 2021–August 2023, frequent nocturia (≥3 voids/night) was associated with higher odds of clinically relevant depressive symptoms in the fully adjusted complex survey model (OR 1.83; 95% CI 1.40–2.40; *p* < 0.001). The association also showed an ordinal trend, supporting nocturia frequency as a clinically meaningful marker rather than a simple binary symptom. These findings extend earlier NHANES-based evidence on lower urinary tract symptoms and depression by focusing specifically on nocturia frequency and by incorporating a recent post-pandemic survey cycle.

These results are consistent with prior studies linking lower urinary tract symptoms, including nocturia, to depressive symptoms. Participants with frequent nocturia were older, had lower educational attainment (20), and more frequently reported smoking, obesity, diabetes, and kidney dysfunction. Sleep disruption (23) and higher high-sensitivity C-reactive protein levels were also more common among those with frequent nocturia (24), supporting a potential mechanistic pathway involving sleep fragmentation and systemic inflammation (25). Notably, while Park et al. (20) reported similar demographic associations, the lack of significant gender differences in our analysis differs from some earlier findings (7), potentially reflecting differences in sample composition or self-reporting biases inherent in NHANES data.

The Chinese exploratory outpatient cohort provided complementary but limited evidence for a possible sleep-related statistical pathway. In this cohort, nocturia frequency was associated with poorer total PSQI scores, and the age-adjusted unstandardized indirect effect through sleep quality was positive. After excluding the PSQI sleep-disturbance component, which contains the nocturnal bathroom-use item, the indirect association was attenuated but remained positive. Nevertheless, PHQ-9 scores did not differ significantly across nocturia groups, and both the total and direct effects were non-significant. Moreover, self-reported nocturia frequency does not distinguish bladder-driven awakenings from voiding that occurs after awakenings attributable to other sleep-disrupting conditions, such as insomnia, mood or anxiety symptoms, pain, or sleep-disordered breathing. This distinction is important because the latter scenario may reflect shared upstream sleep disturbance rather than nocturia-induced sleep fragmentation. Accordingly, the Chinese outpatient data should be interpreted as exploratory evidence for a possible sleep-related statistical pathway, rather than as confirmatory mediation or population-level replication of the NHANES effect estimate.

The age-stratified findings should be interpreted cautiously. The age-stratified pattern was retained as an exploratory finding because multiple subgroup comparisons were performed. Among younger adults, nocturia tended to be associated with higher odds of depression, suggesting that nocturnal voiding may be particularly disruptive when it occurs earlier in adulthood, when it is less expected and may interfere with work, social functioning, and perceived health (24). In contrast, the inverse point estimate for one void/night among adults aged ≥65 years should not be considered evidence of a protective effect (26). One possible explanation is that mild nocturia may be perceived as a common age-related symptom and may be less burdensome than more frequent nocturia, as population-based evidence suggests that ≥2 nocturnal voids are more consistently associated with bother and impaired quality of life (27). However, several non-causal explanations are also plausible. First, the zero-void reference group in older adults may be heterogeneous; older individuals reporting no nocturia may differ in hydration status, mobility, cognitive function, symptom awareness, or medication exposure (28). Second, residual confounding by diuretics, anticholinergic drugs, and treatments for benign prostatic hyperplasia or overactive bladder could not be fully addressed. Finally, the age-stratified estimate may partly reflect statistical variability.

Taken together, these results support greater clinical attention to the coexistence of nocturia, sleep disturbance, and depressive symptoms. The findings do not imply that treating nocturia will prevent depression, nor do they establish temporal direction. Nevertheless, frequent nocturia may help identify individuals who would benefit from broader assessment of sleep quality, psychological symptoms, comorbid disease, and medication exposure. This perspective is especially relevant because nocturia is common, often underreported, and potentially modifiable when driven by sleep disorders, evening fluid intake, metabolic disease, or lower urinary tract conditions.

Several limitations should be acknowledged. First, the cross-sectional design precludes temporal or causal inference and cannot exclude reverse or bidirectional relationships. In particular, self-reported nocturnal voiding may capture awakening-first nocturnal voiding, in which participants wake for reasons other than urinary urgency and then void while awake. Second, nocturia frequency, depressive symptoms, and sleep duration were self-reported and may be affected by recall or classification error; objective measures such as bladder diaries, actigraphy, polysomnography, and clinician-confirmed psychiatric diagnoses were unavailable. Third, despite adjustment for multiple covariates, residual confounding may remain because obstructive sleep apnea severity, anxiety disorders, menopausal status, diuretic use and timing, medications for benign prostatic hyperplasia, overactive bladder or depression, evening fluid and caffeine intake, and detailed lower urinary tract pathology were not assessed. Fourth, the standard PSQI includes sleep disturbance due to getting up to use the bathroom; although sensitivity analysis excluding the sleep-disturbance component yielded a smaller positive indirect effect, measurement overlap remains an important limitation. In addition, the PHQ-9 includes a sleep-related item. Because item-level PHQ-9 data were unavailable in the Chinese exploratory cohort, we could not repeat the indirect-effect analysis after excluding this item; residual content overlap between sleep-quality and depressive-symptom measures may therefore have influenced the exploratory pathway estimates. Fifth, the Chinese cohort was smaller, younger, based on voluntary participation among urology outpatients, and not population representative; cultural, demographic, and healthcare differences limit direct comparability with NHANES. Finally, subgroup analyses involved multiple comparisons and should be interpreted cautiously.

## 5 Conclusion

In conclusion, frequent nocturia was associated with clinically relevant depressive symptoms in U.S. adults after covariate adjustment and incorporation of the NHANES complex survey design, with evidence of an ordinal trend. The age-related heterogeneity observed in subgroup analyses should be regarded as exploratory. The Chinese exploratory outpatient cohort provided exploratory support for a possible sleep-related statistical pathway, and the indirect association was attenuated but remained positive after excluding the PSQI sleep-disturbance component. These findings highlight nocturia as a clinically relevant symptom at the intersection of urinary, sleep, and mental health. Future longitudinal studies incorporating bladder diaries, detailed sleep-disorder assessment, medication data, and PSQI and PHQ-9 item-level or component-specific analyses are needed to distinguish bladder-driven nocturnal awakening from awakening-first nocturnal voiding and to clarify temporal and causal relationships.

## Data Availability

Publicly available datasets were analyzed in this study. The NHANES 2021-2023 data are available from the National Center for Health Statistics at https://wwwn.cdc.gov/nchs/nhanes/continuousnhanes/default.aspx?Cycle=2021-2023. The de-identified data from the Chinese exploratory outpatient cohort are available from the corresponding author upon reasonable request, subject to ethical and institutional requirements.
